# Supplementary material for: Active site specificity profiling datasets of matrix metalloproteinases (MMPs) 1, 2, 3, 7, 8, 9, 12, 13 and 14
Source: Data Brief. 2016 Feb 22;7:299–310. doi: 10.1016/j.dib.2016.02.036 (PMC4777984; doi:10.1016/j.dib.2016.02.036)
Supplement: Supplementary file 10 — Supplementary material [file mmc10.zip › WebPICS_hMMP14_G_1%/P2prime.html]

 

PICS results


|  |  |
| --- | --- |
| **P2prime\_C**  4 in 213 sites   1.9 %    effects > 10 perc. pnts.  (vice-versa in brackets)  P2\_N: 42.5 (10.6) |  |
| **P2prime\_F**  13 in 213 sites   6.1 %    effects > 10 perc. pnts.  (vice-versa in brackets)  P3\_H: 13.1 (33.9)   P2\_Y: 19.3 (31.4)   P1prime\_F: 10.2 (12.1)   P1prime\_M: 16.1 (13.9) |  |
| **P2prime\_K**  52 in 213 sites   24.4 %    effects > 10 perc. pnts.  (vice-versa in brackets)  P3\_V: 12.3 (23.7)   P1\_A: 11.4 (18.0) |  |
| **P2prime\_L**  30 in 213 sites   14.1 %    effects > 10 perc. pnts.  (vice-versa in brackets)  P3\_A: 17.3 (15.3)   P2\_Q: 11.5 (31.4)   P1prime\_I: 23.1 (19.2) |  |
| **P2prime\_M**  7 in 213 sites   3.3 %    effects > 10 perc. pnts.  (vice-versa in brackets)  P1\_C: 11.5 (13.4)   P1\_P: 23.4 (14.9) |  |
| **P2prime\_N**  10 in 213 sites   4.7 %    effects > 10 perc. pnts.  (vice-versa in brackets)  P3\_I: 42.5 (26.5)   P1\_H: 14.8 (13.5)   P3prime\_V: 28.3 (11.3) |  |
| **P2prime\_R**  15 in 213 sites   7.0 %    effects > 10 perc. pnts.  (vice-versa in brackets)  P1prime\_F: 14.8 (20.3)   P3prime\_N: 14.4 (18.0) |  |
| **P2prime\_V**  17 in 213 sites   8.0 %    effects > 10 perc. pnts.  (vice-versa in brackets)  P3prime\_L: 20.7 (11.4)   P3prime\_S: 16.9 (20.6) |  |
